# Performance of Malnutrition Universal Screening Tool and Patient-Generated Subjective Global Assessment in screening for cancer-related malnutrition in Nairobi, Kenya

**DOI:** 10.12688/f1000research.123059.2

**Published:** 2023-11-07

**Authors:** Caroline M.N. Auma, Marshal M. Mweu, Rose O. Opiyo

**Affiliations:** 1 Department of Public and Global Health, Faculty of Health Sciences, University of Nairobi, Nairobi, Kenya

**Keywords:** Bayesian Latent Class Model, Malnutrition, Malnutrition Universal Screening Tool, Patient Generated Global Subjective Assessment, Test accuracy, Cancer patients.

## Abstract

**Background:**

Malnutrition is a common feature among oncology patients. It is responsible for poor response and tolerance to anticancer therapy, increased morbidity, and mortality. More than half of malnourished cancer patients remain undetected owing to lack of effective screening. Body mass index is the main indicator for assessing malnutrition in Kenyan public hospitals. However, it underestimates weight loss in patients with chronic illnesses. The Malnutrition Universal Screening Tool and Patient-Generated Subjective Global Assessment have been widely used in research and clinical practice and have both reported good validity and reliability. However, their diagnostic evaluation has not been performed in Kenya.

**Methods:**

A cross-sectional study was conducted among 138 and 76 cancer outpatients from Kenyatta National Hospital and Texas cancer treatment centres, respectively. Participants had a confirmed disease, stage 1-4 cancer, and aged 18 years and above. They were screened for malnutrition using both Malnutrition Universal Screening Tool and Patient Generated-Subjective Global Assessment. A separate study questionnaire was utilized to gather participant’s socio-demographic and clinical characteristics. A Bayesian latent class modelling framework was employed to infer the tests’ estimates based on participants ‘cumulative scores from the two tests.

**Results:**

The cut-off value of ≥ 1 and ≥ 4 gave the best combination of sensitivity and specificity of Malnutrition Universal Screening Tool and Patient Generated-Subjective Global Assessment. Both tests yielded statistically similar sensitivities and specificities. Predictive values were comparable between the tests and across the two populations. The posterior median true prevalences of malnutrition were high (˃ 54%) and numerically similar between the studied populations.

**Conclusions:**

The performance of both tests among patients with cancer is similar. Healthcare workers are therefore at liberty to use either of them to inform treatment. Given the high true prevalence of cancer-related malnutrition, routine screening is critical and should be included as part of cancer care.

## Introduction

Malnutrition is a common feature in oncology patients. ^1^ Globally, malnutrition affects about 39% to 87% of patients with cancer ^2^ depending on the assessment method ^3^ and the type of cancer involved. ^4^ For example, while breast cancer patients have a low risk of weight loss and subsequent malnutrition, patients with gastrointestinal, lung, and head and neck tumours have a high risk. ^5^ In Kenya, 31% of patients with cancer are malnourished and the greatest burden lies among patients with gastrointestinal tumours at 49.1%. ^6^

Cancer-induced malnutrition has significant clinical and economic burden. The malnutrition in these patients is associated with reduced responsiveness and tolerance to treatment, impaired immune function, increased risk of complications, reduced survival and quality of life, and high healthcare expenditures. ^4^^,^^7^^,^^8^ Globally, 50% of patients dying from cancer are malnourished. ^9^ About 20% of deaths in patients with cancer occur due to malnutrition-related complications rather than the direct effect of malignancy. ^10^ Despite its high prevalence and adverse sequelae in cancer patients, malnutrition in more than half of these patients remains undetected and untreated owing to lack of effective screening. ^11^^–^^13^

Timely recognition of malnutrition is critical for appropriate and intensive nutrition support ^12^ to stop or counteract weight loss and subsequent malnutrition. ^14^^,^^15^ Currently, body mass index (BMI) is the major indicator for the assessment of malnutrition in Kenyan public hospitals. ^16^^,^^17^ However, BMI when used independently is a less sensitive parameter in predicting malnutrition due to large tumours ^9^ and short term fluid imbalance. ^18^^,^^19^ Therefore, patients can suffer malnutrition regardless of BMI value because loss in lean body mass is masked by ascites and body fat. ^20^^,^^21^ The commonly available screening tools for use in oncology setting are Patient Generated-Subjective Global Assessment (PG-SGA) and Malnutrition Universal Screening Tool (MUST). These screening tools assess patients’ nutrition status using a cluster of two or more nutrition-related parameters, thus are superior to BMI. ^4^^,^^19^^,^^20^ PG-SGA and MUST have been widely used in both clinical practice ^22^^,^^23^ and research. ^24^ They have reported good sensitivity (Se) and specificity (Sp) in identifying the nutrition status of patients with cancer. ^25^^–^^30^

The PG-SGA is a simple method employed in assessment of nutritional risk and identification of those who would benefit from nutritional support. For this reason, it is considered the most appropriate tool for detecting malnutrition in cancer patients. ^31^ The PG-SGA is a 4-in-1 instrument to screen, assess, and monitor malnutrition and its risk factors and to triage for interdisciplinary intervention. ^25^^,^^32^ It is inexpensive, quick to complete (10-15 minutes), ^22^^,^^33^ and user-friendly. ^34^^,^^35^ More to its merits, the first four boxes can be completed by a patient, which empowers them on matters related to their health and reduces the total time involved. In addition, PG-SGA contains more nutrition impact symptoms that are vital to cancer patients. ^18^ Lastly, PG-SGA assess nutrition status on a continuous scale, which allows it to detect subtle changes in nutritional status occurring over a short period and triage patients for appropriate nutrition intervention. ^25^^,^^31^^,^^36^ Overall, PG-SGA allows malnourished patients to be quickly identified and prioritized for care, thus a suitable screening tool. ^22^^,^^25^

The MUST is designed to categorize adults as either malnourished, obese, or at risk of malnutrition and identify those who would benefit from appropriate nutrition intervention. ^37^ It does not incorporate subjective/clinical parameters reflecting changes in nutritional risk and status and neither does it require time-consuming calculations. ^23^ It is quick to complete (3 – 5 minutes). ^38^ It is also inexpensive, easy to apply and interpret, and hence acceptable to both patients and healthcare workers. ^23^^,^^38^^,^^39^ MUST combines weight loss and BMI, which reduces misclassification errors, thus improving Se and Sp. ^19^ Additionally, a patient can screen him/herself without the help of a health worker with a time response as low as 5 minutes. ^40^

Although these tools have been extensively validated in various countries, their performance has not been evaluated in Kenya. Additionally, in countries where they have been evaluated, evaluations were done against reference standards such as Subjective Global Assessment, ^25^^,^^26^ Mini-Nutritional Assessment, ^27^ Nutritional-Risk Screening-2002, ^41^ Albumin and BMI. ^42^ This may have plagued the estimates with information and selection bias. ^43^ Nevertheless, the accuracy of two or more tests can be quantified without prior knowledge of the true disease status by using latent class models. ^43^^,^^44^ Thus, the objective of the present study was to derive the Se and Sp together with the positive and negative predictive values of PG-SGA and MUST for malnutrition in head and neck, respiratory, and gastrointestinal cancer patients using the Bayesian Latent Class Model (BLCM).

## Methods

### Study design and setting

A facility-based cross-sectional study was conducted to evaluate the performance of the MUST and PG-SGA in screening for malnutrition among cancer patients. This study design was most suitable due to the ease of recruitment of cancer patients presenting to the cancer clinic and its suitability for the descriptive nature of the study.

The study was conducted at Kenyatta National Hospital-Cancer Treatment Centre (KNH-CTC) and Texas Cancer Centre (TCC) in Nairobi, Kenya. KNH is the largest national public referral hospital in Kenya. It registers 80,000 inpatients and 500,000 outpatients annually. It offers comprehensive cancer services, which include diagnosis and treatment (chemotherapy, radiotherapy, surgical and hormonal therapy). The three most commonly diagnosed carcinomas are cervical, breast, and oesophageal cancer in that order. KNH-CTC provides both inpatient and outpatient services. Although nutrition screening of cancer patients is not conducted routinely, there is an in-house nutritionist whose services are sought under certain circumstances.

The TCC is the leading private cancer care and treatment centre in Kenya. It receives approximately 11000 patients annually. Apart from cancer diagnosis and treatment, TCC offers pain and palliative care services, counselling, physiotherapy, and rehabilitation services. With regards to nutrition services, currently, TCC does not have an in-house nutritionist, but under certain circumstances, one is outsourced. Consequently, nurses and doctors offering oncological services provide nutrition support. Weight and height are the measurements taken for BMI scoring but not routinely.

### Study population, eligibility and selection of participants

The study population consisted of all patients with head and neck, respiratory and gastrointestinal cancers attending outpatient oncology clinic at KNH-CTC and TCC. On eligibility, a patient had to have established disease, that is, stage 1-4 cancer, aged 18 years and above, physically stable, and have given informed consent. To obtain the study sample, a simple random sampling technique was applied to a sampling frame comprising of patients booked for evaluation in each facility on a given day. To ascertain the number of patients to be sampled from each facility, a probability proportional to size sampling ^45^ was employed guided by the number of patients seen in a given facility in a particular month. As such, 138 and 76 cancer patients from KNH-CTC and TCC, respectively, were recruited.

### Study procedures and outcome assessment

Two research assistants with a medical background who had previously been trained on interviewing techniques recruited and interviewed participants. Upon obtaining informed consent from the participants, the MUST and PG-SGA (either English or Kiswahili version depending on the individual’s preference) were administered through a face-to-face interview in a private room within the Cancer Clinic. The MUST and PG-SGA tools were completed just before provision of oncology services. Aside from the nutrition status, the patients’ socio-demographic characteristics (age, sex, marital status, level of education, area of residence and employment status) and clinical characteristics (tumour site and cancer stage) were also recorded.

### Sample size determination

The required sample size was estimated using McNemar’s sample size formula for paired proportions. ^46^

$$n_{\mathrm{per}\;\text{test}}={\left(\frac{Z_{\alpha /2}\sqrt{P_{\text{disc}}}+Z_{\beta }\sqrt{P_{\text{disc}}-{P^{2}}_{\text{diff}}}}{P_{\text{diff}}}\right)}^{2}$$

$$P_{\text{disc}}=\left(1-{\mathrm{Se}}_{1}\right)+\left(1-{\mathrm{Se}}_{2}\right)$$

$$P_{\text{diff}}=\left(1-{\mathrm{Se}}_{1}\right)-\left(1-{\mathrm{Se}}_{2}\right)$$

where: $n_{\mathrm{per}\;\text{test}}$ was the sample size required for each test, $Z_{\alpha /2}$ (1.96) was the critical value for specifying the two tailed 95% confidence level, $Z_{\beta }$ (-0.84) was the critical value specifying the statistical power of 80% desired, ${\mathrm{Se}}_{1}$ and ${\mathrm{Se}}_{2}$ were sensitivity estimates of the MUST and PG-SGA, respectively, from literature. Notably, ${\mathrm{Se}}_{1}$ was set at 0.8 ^28^ and ${\mathrm{Se}}_{2}$ set at 0.98. ^25^ Based on the specified figures above, a total sample size of 214 was reached after adjusting upwards by 5% to account for non-response.

### Ethical consideration

The study participants provided written informed consent prior to engaging in the study. Approval to conduct the study was granted by Kenyatta National Hospital and University of Nairobi joint Ethics and Research Committee (KNH-ERC/A/315).

### Target condition

This referred to the latent (unobserved) malnutrition status regardless of the severity level among head and neck, respiratory and gastrointestinal cancer patients that is targeted by MUST and PG-SGA.

### Malnutrition Universal Screening Tool

The MUST employs three stand-alone criteria to classify patients’ nutritional status: BMI score (BMI ˃20 kg/m ^2^ = 0, BMI 18.5-20 kg/m ^2^ = 1, BMI <18.5 kg/m ^2^ = 2), unintentional weight loss score in the last 3 to 6 months (weight loss < 5% = 0, weight loss 5-10% =1, weight loss ˃ 10% = 2) and acute disease effect score (add a score of 2 if there has been or is likely to be no nutrition intake for > 5 days). Each parameter is scored on a scale of 0, 1, or 2 giving an overall total score of six. Overall risk of malnutrition is classified as low risk if the score = 0, moderate risk if the score = 1, and high risk if the score is ≥ 2. ^23^ In this study, a patient with a total score of ≥1 was considered malnourished.

### Patient-Generated Subjective Global Assessment

The PG-SGA relies on weight history, changes in patient’s dietary intake, presence of gastrointestinal symptoms, functionality, diagnosis, age, metabolic stress, and physical examination (subcutaneous fat loss, muscle wasting, and fluid status) to assess a patient’s nutritional risk. For each of the above-mentioned components, 0-4 points are awarded based on the relative impact on nutritional status. The overall total scores of PG-SGA range from 0-52. Based on the scores, patients were divided into three categories: (PG-SGA < 2, well-nourished/no risk), (PG-SGA ≥ 2 and < 9 suspected/moderate risk), (PG-SGA ≥ 9, severe risk). Three potential cut-offs were examined: individuals being categorised as malnourished if they had scores ≥2, ^9^^,^^26^ ≥4, ^42^^,^^47^ ≥9. ^25^^,^^48^

### Statistical analysis

A Bayesian modelling framework was used to derive the prevalence, Se and Sp estimates along with the predictive values of the tests. The BLCM was fitted in OpenBUGS software (v 3.2.2) ^49^ but called from R (v. 3.6.2) via the Brugs package (v0.9-0). ^50^ Importantly, the model design and reporting were guided by the standards for reporting of diagnostic accuracy studies that use BLCMs (STARD-BLCM). ^51^ The Bayesian code is available as *Underlying data*. ^52^

According to Hui and Walter, ^44^ the BLCM has the following assumptions: (i) there must be two or more populations with differing prevalences. For this study, two populations of patients attending KNH-CTC and TCC facilities were established. Owing to the inherent socio-economic differences between the facilities’ catchment populations, the prevalences of cancer-related malnutrition were presumed to be distinct. (ii) The Se and Sp of both tools should be consistent across the populations. (iii) Given the disease status, there was conditional independence between the two tests. This was a reasonable assumption considering the two tools have separate symptom targets: MUST targets changes in body composition thus, assesses chronic malnutrition whereas PG-SGA assesses gastrointestinal symptoms preventing food intake thus, targets acute malnutrition. ^53^ Consequently, the probability of a patient testing either positive or negative to one test is not affected by the result of the other test.

It was assumed that the different combinations of test results, for each population, observed as counts $\left(O_{k}\right)$ follow a multinomial distribution:

$$O_{k}|{\mathrm{Se}}_{i}{\mathrm{Sp}}_{i}p_{k}∼\text{mulitinomial}\;{\text{prob}}_{k}n_{k}$$

Where ${\mathrm{Se}}_{i}\;\text{and}\;{\mathrm{Sp}}_{i}$ are the *Se* and *Sp* measures for the $i^{\mathrm{th}}\;$ test (i=1, 2) and $p_{k}$ represent the $k^{\mathrm{th}}$ population’s prevalence. ${\text{Prob}}_{k}$ , represents a vector of probabilities of observing the tests’ results’ combinations (such as +, +), while $n_{k}$ is the sample size used in population k. The probabilities are defined using the specific test characteristics ( *Se* and *Sp*) and prevalence ( *p*) of each population. For example, ${\text{prob}}_{1}\;$ for a person who tests positive to both tests is given by:

$${\text{prob}}_{1}=P_{r}\left({T_{1}}^{+}{T_{2}}^{+}|D^{+}\right)+P_{r}\left({T_{1}}^{+}{T_{2}}^{+}|D^{-}\right)={\mathrm{Se}}_{1}{\mathrm{Se}}_{2}P_{1}+\left[1-{\mathrm{Sp}}_{1}\right]\left[1-{\mathrm{Sp}}_{2}\right]\left[1-P_{1}\right]$$

Since there were two populations, the Latent Class Model contained six parameters: each test’s *Se* and *Sp*, as well as two population-specific prevalences. These six parameters were estimated from six degrees of freedom obtained from each of the two populations. As there was no reliable prior information on any of the parameters, uninformative priors (beta (1, 1)) were used.

The Positive Predictive Value (PPV) and Negative Predictive Value (NPV) for each test within population k were derived using the following formula:

$$\mathrm{PPV}=P_{K}{\mathrm{Se}}_{i}/\left(P_{k}{\mathrm{Se}}_{i}+\left[1-P_{k}\right]\left[1-{\mathrm{SP}}_{i}\right]\right)$$

$$\mathrm{NPV}=\left[1-P_{K}\right]{\mathrm{Sp}}_{i}/\left(P_{k}\left[1-{\mathrm{Se}}_{i}\right]+\left[1-P_{k}\right]{\mathrm{Sp}}_{i}\right)$$

To select the most optimal cut-off values, Youden indices ^54^ were computed using the Se and Sp obtained at each of the following pairs of cut-off values: ≥1 for MUST paired with each of the following PG-SGA cut-off values (≥2, ≥4, and ≥9). The pair of cut-off points with the highest Youden index was chosen.

Two Markov Chain Monte Carlo (MCMC) chains with varying values were used to initialize the model. We ran 6000 iterations of the model with the initial 3500 discarded as the burn-in phase. Convergence of the MCMC chain was assessed by visual appraisal of the density plots and Gelman-Rubin Diagnostic plots. The posterior distribution of prevalence of each of the two populations, the test estimates, and their respective predictive values were reported as the median and their associated 95% posterior credible intervals (PCI).

## Results

### Socio-demographic and clinical characteristics of study participants

A total of 214 participants were selected, and 202 consented to participate, giving a response rate of 94.4%. However, of the 202 participants, 14 were excluded from analysis due to lack of records on previous anthropometric measurements, which rendered their total scores from MUST and PG-SGA unreliable.

The socio-demographic and clinical features of 188 participants are displayed in Table 1. Notably, the participants’ median age was 56.5 years (range: 18-81 years). Majority of the participants were males (64.36%). Only 46% of the participants had attained at least a secondary school certification. Clinically, patients with gastrointestinal cancers formed the largest proportion of the sample at 54.26%. Approximately 34% of the participants had stage 4 cancer.

**Table 1.** Summary statistics of socio-demographic and clinical characteristics of study participants from Kenyatta National Hospital-Cancer Treatment Centre (KNH-CTC) and Texas Cancer Centre (TCC), Kenya, (n = 188).

Variable	Values	Median	Range	Frequency n (%)
**Age (in completed years)**				
Overall (N = 188)	**-**	56.5	18-81	**-**
KNH-CTC (n = 126)	**-**	56	19-80	**-**
TCC (n = 62)	-	57.5	18-81	-
**Sex**				
Overall	Male	-	-	121 (64.36)
	Female	-	-	67 (35.64)
KNH-CTC	Male	-	-	81 (64.21)
	Female	-	-	45 (35.71)
TCC	Male	-	-	40 (64.52)
	Female	-	-	22 (35.48)
**Marital status**				
Overall	Single	-	-	17 (9.04)
	Married	-	-	145 (77.13)
	Separated/divorced	-	-	8 (4.26)
	Widowed	-	-	18 (9.57)
KNH-CTC	Single	-	-	10 (7.94)
	Married	-	-	99 (78.57)
	Separated/divorced	-	-	7 (5.56)
	Widowed	-	-	10 (7.93)
TCC	Single	-	-	7 (11.29)
	Married	-	-	46 (74.19)
	Separated/divorced	-	-	1 (1.61)
	Widowed	-	-	8 (12.90)
**Level of education**				
Overall	No formal education	-	-	15 (7.98)
	Primary	-	-	86 (45.74)
	Secondary	-	-	63 (33.51)
	Tertiary	-	-	24 (12.77)
KNH-CTC	No formal education	-	-	9 (7.14)
	Primary	-	-	60 (47.62)
	Secondary	-	-	44 (34.92)
	Tertiary	-	-	13 (10.32)
TCC	No formal education	-	-	6 (9.68)
	Primary	-	-	26 (41.94)
	Secondary	-	-	19 (30.65)
	Tertiary	-	-	11 (17.74)
**Area of residence**				
Overall	Nairobi		-	29 (15.43)
	Other		-	97 (84.57)
**Employment status**				
Overall	Employed	-	-	64 (34.04)
	Unemployed	-	-	124 (65.96)
KNH-CTC	Employed	-	-	39 (30.95)
	Unemployed	-	-	87 (69.05)
TCC	Employed	-	-	25 (40.32)
	Unemployed	-	-	37 (59.68)
**Tumour location**				
Overall	Gastrointestinal			102 (54.26)
	Head and neck	-	-	67 (35.64)
	Respiratory	-	-	19 (10.11)
KNH-CTC	Gastrointestinal	-	-	71 (56.35)
	Head and neck	-	-	43 (34.13)
	Respiratory	-	-	12 (9.52)
TCC	Gastrointestinal	-	-	31 (50.00)
	Head and neck	-	-	24 (38.71)
	Respiratory	-	-	7 (11.29)
**Stage of cancer disease**				
Overall	Stage I	-	-	23 (12.23)
	Stage II	-	-	46 (24.47)
	Stage III	-	-	55 (29.26)
	Stage IV	-	-	64 (34.04)
KNH-CTC	Stage I	-	-	19 (15.07)
	Stage II	-	-	35 (27.78)
	Stage III	-	-	35 (27.78)
	Stage IV	-	-	37 (29.37)
TCC	Stage I	-	-	4 (6.45)
	Stage II	-	-	11 (17.74)
	Stage III	-	-	20 (32.36)
	Stage IV	-	-	27 (43.55)

### Test outcomes, sensitivity and specificity

The cross-classified counts of the two test results at various cut-off points are presented in Table 2. The cut-off value of ≥1 for MUST and ≥4 for PG-SGA gave the best combination of the Se and Sp (see Table 3). Thus, this cut-off was used to infer subsequent parameters. The PG-SGA registered a Se of (92.4; 95% PCI [81.2; 99.6]) and Sp of (72.5% 95% PCI [54; 97.2]) while MUST had a Se of (83.1 95% PCI [67.4; 98.9]) and a Sp (85.7; 95% PCI [71.4; 99.6]). Although the Se and Sp of the two tests differed numerically, they were statistically similar based on 95% PCIs.

**Table 2.** Cross-classified counts by population for MUST and PG-SGA tests for screening for malnutrition among cancer patients seeking outpatient oncological care at Kenyatta National Hospital-Cancer Treatment Centre (KNH-CTC) and Texas Cancer Centre (TCC), Kenya during the period October-November 2021.

Population	Cut point	Test outcome (MUST/PG-SGA)				
	MUST/PG-SGA	(+ ^a^/+)	(+/- ^b^)	(-/+)	(-/-)	Total (%)
KNH-CTC	≥1, ≥2	65	3	40	18	126 (67%)
TCC		32	1	25	4	62 (33%)
KNH-CTC	≥1, ≥4	61	7	23	35	126 (67%)
TCC		26	7	12	17	62 (33%)
KNH-CTC	≥1, ≥9	33	35	6	52	126 (67%)
TCC		16	17	4	25	62 (33%)

^a^ Positive.

^b^ Negative.

**Table 3.** Estimates of sensitivity and specificity of MUST and PG-SGA at different pair of cut-off values and Youden indices for the best cut-off point.

Cut-off Value		Test parameter	Estimate (95% PCI)	Y.index _MUST_	Y.index _PG-SGA_
MUST	PG-SGA				
≥1	≥2	Se _MUST_	11.7 (0.7; 32.6)	-55.38	-49.9
		Sp _MUST_	46.6 (3.4; 77.6)		
		Se _PG-SGA_	33.7(5.6; 45)		
		Sp _PG-SGA_	2.8 (0.15; 8.7)		
≥1	≥4	Se _MUST_	83.1 (67.4; 98.9)	69.2	65.0
		Sp _MUST_	85.7 (71.4; 99.6)		
		Se _PG-SGA_	92.4 (81.2; 99.6)		
		Sp _PG-SGA_	72.5 (54; 97.2)		
≥1	≥9	Se _MUST_	88.9 (74.9; 99.3)	61.07	58.7
		Sp _MUST_	71.3 (55.5; 97.5)		
		Se _PG-SGA_	66.2 (43.9; 97.1)		
		Sp _PG-SGA_	92.9 (82.9; 99.6)		

### Predictive values and prevalences

On predictive values, NPVs and PPVs were statistically similar between the tests and across the two populations ( Table 4). In KNH, MUST had a NPV of 77.9% and a PPV of 89.7% while PG-SGA had a NPV of 87.2% and a PPV of 82.9%. In TCC, MUST had a NPV of 81.2% and a PPV of 87.8% while PG-SGA a NPV of 89.2% and a PPV of 80.1%. The posterior median true prevalences of malnutrition were high (˃54%) and numerically similar between the studied populations ( Table 4).

**Table 4.** Estimates of positive and negative predictive values of MUST and PG-SGA and Prevalences across Kenyatta National Hospital-Cancer Treatment Centre (KNH-CTC) and Texas Cancer Centre (TCC). PPV=Positive Predictive Value; NPV=Negative Predictive Value.

Location	Test parameter	Estimate (95% PCI	
		MUST	PG-SGA
KNH	NPV	77.9 (49.8; 98.9)	87.2 (65.2; 99.3)
	PPV	89.7 (73.7; 99.4)	82.9 (62.5; 99.1)
	**Prevalence**	**59.1 (41.9; 75.6)**	
TCC	NPV	81.2 (50.9; 99.1)	89.2 (64.2; 99.6)
	PPV	87.8 (65.6; 99.4)	80.1 (54.2; 98.9)
	**Prevalence**	**54.6 (32.8; 75.7)**	

### Convergence of diagnostic plots

On convergence of the MCMC chains, the density plots of all the posterior estimates yielded unimodal distributions. Similarly, the Gelman-Rubin diagnostic statistics for the parameters produced unitary ratios. These results suggest convergence of the chains.

## Discussion

### Sensitivity and specificity of the MUST and PG-SGA in screening for malnutrition

In this study, MUST registered a Se of 83.1% supported by finding from other studies ^28^^,^^29^ and a Sp of 85.7% in agreement with findings from previous studies. ^28^^,^^30^ However, in the literature, the Se of MUST has been shown to vary from 29% ^55^ to 97% ^41^ similar to its Sp from 48.9% ^56^ to 94.5%. ^29^ This inconsistency can be logically explained by the percentage weight loss, which is a crucial parameter in the MUST tool. ^28^ For example, patients may have lost weight in the past, but their present weight recovery was not calculated since MUST does not give this provision. ^56^ In addition, contrary to this study, in those studies, estimation of the test accuracy was based on the assumption that a perfect reference standard existed. This could have led to biased estimates. More so, MUST in its categorization of nutrition status, relies on the percentage of weight lost between 3 and 6 months. As a result, where patients were required to self-report the involuntary weight loss, there was a possibility of under-estimation hence affecting Sp. This is supported by a study conducted in Belgium, which reflected the propensity for underestimation of weight loss in oncology patients. ^57^ In this study, all patients with no previous records of anthropometric measurements were excluded from the analysis. Although MUST was found to be a suitable screening tool, it requires that patients be weighed for the determination of BMI and weight loss. Thus, inaccurate weight in patients with oedema or ascites could lead to underestimation of nutritional risk in some patients. ^33^

The PG-SGA yielded a Se and Sp of 92.4% and 72.5%, respectively. The findings demonstrate that the PG-SGA is a highly sensitive malnutrition screening tool in the outpatient oncology population, consistent with findings from previous studies. ^25^^–^^27^^,^^42^ An ideal screening tool would be 100% sensitive and specific. But, the necessity to categorize all malnourished patients (Se) correctly takes precedence over misclassification of well-nourished individuals (Sp). ^58^ The Sp of PG-SGA varies greatly in published studies, with estimates ranging from 2.3% ^1^ to 88.1%. ^27^ The Sp of 72.5% observed in this study falls within the above range. The variability could be due to three things: First, use of different reference standards in deriving test estimates, which was not the case in this study, for example, specificities of 2.3% and 88.1% were based on albumin and Mini-Nutrition Assessment tool, respectively. Secondly, different administration methods, since the score from participant-versus researcher administered may not be comparable. This is because, the authors of PG-SGA speculate that patients may over-report symptoms based solely on their presence, regardless of their impact on food intake, ^58^ thus underestimating Sp. For example, Sp of 21.8% ^59^ and 82% ^25^ were derived from patient and researcher administered studies, respectively. This study was researcher administered thus a good Sp was registered. Lastly, the scores derived from component Number Three of PG-SGA questionnaire (nutrition impact symptoms and other factors). In this section, any symptom impairing food intake reported by the patient is scored, and all points are cumulative (maximum 24 points). ^22^ This could also rise the number of false positives thus underestimating Sp. However, this wide range of symptoms may help detect more patients at risk of malnutrition. As the detection of symptoms that impair nutritional intake in the early stages of the disease may be advantageous for proactively preventing cancer-related malnutrition. ^58^ The observed high Se and the ability to determine what elements are influencing nutrition status make PG-SGA a suitable nutritional screening tool. However, its accuracy is dependent upon the experience of an observer. ^60^ Therefore, it requires a trained healthcare professional to complete the assessment and score the tool. ^58^

### Predictive values of the MUST and PG-SGA in screening for malnutrition

In this study, the two tests yielded comparable estimates of PPV and NPV across the two populations based on the observed 95% PCIs. Therefore, if each test is used singly to screen for malnutrition, it is reliable and can inform treatment. Hence, the tool of choice is dependent on the purpose of the assessment, prognosis or even on the response to nutritional intervention ^33^ bearing in mind the inherent shortcomings of each.

### True prevalence of malnutrition

The prevalence of malnutrition among patients with cancer was high (˃ 54%). The findings are not unusual since cancer patients, particularly those suffering from head and neck, lung, and gastrointestinal cancer, carry the greatest burden of malnutrition among hospital patients. ^61^

Although the findings of this study are similar to those observed in Spain (50%) ^62^ and Italy (51%), ^63^ higher prevalences have been reported elsewhere. For example, malnutrition was found to be present in 76% of ambulant cancer patients getting radiation therapy in Brazil ^25^ In London 71% of cancer patients were malnourished, ^64^ and in Ethiopia, a prevalence of 90.6% in ambulant cancer patients on treatment was reported. ^65^ The prevalence of cancer-induced malnutrition is often cited as 40-80%, ^5^^,^^66^ which largely depends on assessment method, clinical setting, and case-mix of patients. Notably, the lower and upper PCI limits in this study fall within the quoted range (40-80%). With respect to the parameters of this study, the study focused on specific malignancies, there was no restriction on cancer stage, and the analysis did not take into account whether or not patients received treatment. Moreover, this study used a combination of MUST and PG-SGA to estimate prevalence, unlike the above studies that employed one tool. The above factors could have been the cause of the disparity between the results of this study and the above-mentioned studies.

In Kenya, in contrast to this study, lower prevalences of 31% ^6^ and 13.4% ^17^ have been obtained using the same population. Although lower, 31% falls within the lower PCI limit obtained at TCC in this study. The disparity could be ascribable to the different PG-SGA classification methods used. While this study employed the scoring method, a prevalence of 31% was based on categorical method. The scoring method is more sensitive than the categorical classification, thus capturing more patients and leading to a higher prevalence. ^47^ For example, a study conducted among cancer patients in Norway recorded a Se of 50.0% and 60.7% for PG-SGA categorical and scoring classification methods, respectively. ^47^ In regards to a prevalence of 13.4%, the disparity could be explained by the different assessment methods used. A prevalence of 13.4% ^17^ was based on BMI < 18.5 kg/m ^2^, while in this study it was based on both the PG-SGA and MUST, which are superior to BMI.

The major strength of this study was using a Bayesian Latent Class Modelling framework, which minimized bias in test estimates, as it does not rely on a reference standard. A few limitations are inherent in this study that readers should be aware of while interpreting the findings. First, the PG-SGA is in form of a questionnaire targeting symptoms occurring within two weeks or one month of the time of screening. Therefore, the study participants may have failed to recall accurately, leading to either over-reporting or under-reporting of their symptoms. These may have biased the tool’s Se and Sp. Second, due to the study’s focus on specific cancers, findings may not be generalizable to other ambulant cancer populations. Third, patients who rejected taking part in this study did so due to pain, nausea, and weakness or because they thought, the study was too burdensome. Since these patients were more likely to be malnourished, this could have resulted in sample bias.

## Conclusions

Using the Latent Class analysis, we have estimated the Se, Sp, and predictive values of both PG-SGA and MUST. The two tests achieved an accepted professional standard for Se (≥80%) and yielded good specificities. In respect to predictive values, the two tests produced comparable estimates. As such, healthcare workers are at liberty to use any of the two screening tools. Considering the high true prevalence observed in the two study populations, malnutrition screening among cancer patients should be done at diagnosis, during treatment and follow-up.

## Data availability

### Underlying data

Raw dataset for the study is kept under restricted access since it contains sensitive participant information. Access to the raw data is possible upon placing a formal request to the corresponding author ( carolinemuseka@gmail.com). The replication data and analysis scripts for this manuscript are available from the Harvard Dataverse.

Harvard Dataverse: Performance of Malnutrition Universal Screening Tool and Patient-Generated Global Subjective Assessment in screening for cancer-related malnutrition in Nairobi County, Kenya. https://doi.org/10.7910/DVN/HS5YM1. ^52^

This project contains the following underlying data:

• R_Code_maln.R (Rscript for analysis)
• Maln_data_xlsx (Analysis dataset)
• Questionnaire.pdf (The questionnaire used for data collection)

Data are available under the terms of the Creative Commons Zero “No rights reserved” data waiver (CC0 1.0 Public domain dedication).
