# Supplementary material for: Performance of Malnutrition Universal Screening Tool and Patient-Generated Subjective Global Assessment in screening for cancer-related malnutrition in Nairobi, Kenya
Source: F1000Res. 2023 Nov 7;11:755. Originally published 2022 Jul 7. [Version 2] doi: 10.12688/f1000research.123059.2 (PMC12198732; doi:10.12688/f1000research.123059.2)
Supplement: Peer review history

# Reviewer report: Reviewer response for version 2

**DOI:** 10.5256/f1000research.158459.r383801

**Published:** 2025-06-25

**Authors:** Paul Mwaniki

**Affiliations:** 1 KEMRI-Wellcome Trust Research Programme, Nairobi, Kenya

The study employs bayesian latent class analyses to estimate the sensitivity and specificity of two malnutrition scoring tools in two cancer treatment centers. The authors show that both tools have good sensitivity and specificity. 

 The authors have justified the use of LCA stating that the assumption of conditional independence of the tests given the true malnutrition status is likely to hold given that the tests are composed of different questions.

 Table 1 could be better structured by having the centres as columns with the values being frequencies and percentages for categorical variables and median and range for continuous variables.

Is the work clearly and accurately presented and does it cite the current literature?

Yes

If applicable, is the statistical analysis and its interpretation appropriate?

Yes

Are all the source data underlying the results available to ensure full reproducibility?

Yes

Is the study design appropriate and is the work technically sound?

Yes

Are the conclusions drawn adequately supported by the results?

Yes

Are sufficient details of methods and analysis provided to allow replication by others?

Yes

Reviewer Expertise:

Statistics and Data science

I confirm that I have read this submission and believe that I have an appropriate level of expertise to confirm that it is of an acceptable scientific standard.

# Reviewer report: Reviewer response for version 1

**DOI:** 10.5256/f1000research.135124.r196451

**Published:** 2023-10-26

**Authors:** Mariana Kruger

**Affiliations:** 1 Department of Paediatrics and Child Health, Faculty of Medicine and Health Sciences, Stellenbosch University, Stellenbosch, Western Cape, South Africa

1. Check spelling and edit. Professional grammar editing will improve the readability of the text. Avoid long sentences with high density of information. Check the following and correct: “…. *cute disease effect score*…” under *Malnutrition Universal Screening Tool.*
2. Write out abbreviations when used for the first time e.g., Se and Sp.
3. Indicated clearly under the section “ *Test outcomes, sensitivity and specificity*” what cut-off point was used for which test; either by adding respectively or linking the cut-off point to the test. 
4. Please add the time needed to use both tests as it seems to be quiet time-consuming for screening tests, especially if the PGA-SGA should be done by the treating physician.

Is the work clearly and accurately presented and does it cite the current literature?

Yes

If applicable, is the statistical analysis and its interpretation appropriate?

Yes

Are all the source data underlying the results available to ensure full reproducibility?

Yes

Is the study design appropriate and is the work technically sound?

Yes

Are the conclusions drawn adequately supported by the results?

Yes

Are sufficient details of methods and analysis provided to allow replication by others?

Partly

Reviewer Expertise:

Oncology, nutrition, paediatrics, ethics

I confirm that I have read this submission and believe that I have an appropriate level of expertise to confirm that it is of an acceptable scientific standard.

# Response

**Published:** 2023-11-02

**Authors:** Caroline Auma

**Affiliations:** University of Nairobi, Kenya

**Response to comment 1**

 I confirm that proofreading has been done to correct the grammatical errors.

**Response to comment 2**

 We have included this in the second last line of paragraph 3 in the introduction section: Sensitivity (Se) and Specificity (Sp).

**Response to comment 3**

 We have revised this by linking the cut-off to the test.

 As such, it now reads: The cut-off value of ≥1 for MUST and ≥4 for PG-SGA gave the best combination of the Se and Sp.

**Response to comment 4**

 We have supplied the information on the time needed for the tests: PG-SGA and MUST in paragraph 4 and paragraph 5 of the introduction section, respectively.

# Reviewer report: Reviewer response for version 1

**DOI:** 10.5256/f1000research.135124.r143589

**Published:** 2022-08-15

**Authors:** Oscar Ngesa

**Affiliations:** 1 Department of Mathematics and Physical Sciences, Taita Taveta University, Voi, Kenya

Below is my review of the paper titled “Performance of Malnutrition Universal Screening Tool and Patient-Generated Global Subjective Assessment in screening for cancer-related malnutrition in Nairobi, Kenya”.

 The study aims at evaluating the performance of the Patient Generated-Subjective Global Assessment (PG-SGA) and Malnutrition Universal Screening Tool (MUST) in screening for malnutrition among cancer patients in Kenya. The cancers of interest in this setting are head and neck, respiratory and gastrointestinal cancer. Bayesian Latent Class Model (BLCM) was employed in the analysis. It is a preferred approach in the absence of a gold standard.

 Generally, the paper is well written in an appropriate language and easy to follow. 

 Below are my specific comments and queries.

- In the introduction, put 39% to 87% instead of 39 to 87%.
- All citations at the end of each sentence should be placed before the period/full stop rather than after. This needs to be corrected in the entire document.
- The noun citation styles Kaduka,Bukania,17  and Ramboer, Verhamme57 do not seem to be correct. Confirm the style.
- Can the authors check for model stability and robustness of the estimates via sensitivity analysis? Changing the different priors.
- The authors mentioned that they would assess convergence of the models using visual appraisal of the density plots and Gelman-Rubin Diagnostic plots. However, none of these is reported in the document.

Is the work clearly and accurately presented and does it cite the current literature?

Yes

If applicable, is the statistical analysis and its interpretation appropriate?

Partly

Are all the source data underlying the results available to ensure full reproducibility?

Yes

Is the study design appropriate and is the work technically sound?

Yes

Are the conclusions drawn adequately supported by the results?

Yes

Are sufficient details of methods and analysis provided to allow replication by others?

Partly

Reviewer Expertise:

Statistical Modeling; Bayesian analysis; Malnutrition Analysis

I confirm that I have read this submission and believe that I have an appropriate level of expertise to confirm that it is of an acceptable scientific standard, however I have significant reservations, as outlined above.

# Response

**Published:** 2023-11-02

**Authors:** Caroline Auma

**Affiliations:** University of Nairobi, Kenya

**Response to comment 1**

 Revised as recommended. Now reads 39% to 87%.

**Response to comment 2**

 We have revised this as advised. However, It looks like this is an error from the journal editors because, from the Word document that I submitted, the citations were correctly placed at the end.

**Response to comment 3**

 This has been revised to align with the rest of the in-text citations.

**Response to comment 4**

 Based on the three assumptions of the latent class analysis, we only met the minimum criteria. In particular, we only had two populations thus the constancy of Se and Sp estimates could not be assessed.

 Secondly, the two tests (MUST and PG-SGA) target different symptoms thus we assumed conditional independence.

 Finally, there was no prior information on any of the parameters in particular the Se and Sp. Therefore sensitivity analysis by modifying priors wouldn't be possible.

**Response to comment  5**

 We have included this as the last part of the results section.

 It reads: On the convergence of the MCMC chains, the density plots of all posterior estimates yielded unimodal distributions. Similarly, the G elman-Rubin diagnostic statistics for the parameters produced unitary ratios. These results suggest convergence of the chains.

# Response

**Published:** 2023-11-09

**Authors:** Caroline Auma

**Affiliations:** University of Nairobi, Kenya

More to Comment 2

 Regarding citations coming after the full stops, I noticed the same issue after version 2 was published. I engaged the editors and they said the change couldn't be made due to F1000 journal style.
